# Disentangling transcriptional responses in plant defense against arthropod herbivores

**DOI:** 10.1038/s41598-021-92468-6

**Published:** 2021-06-21

**Authors:** Alejandro Garcia, M. Estrella Santamaria, Isabel Diaz, Manuel Martinez

**Affiliations:** 1 Centro de Biotecnología y Genómica de Plantas, Universidad Politécnica de Madrid – Instituto Nacional de Investigación y Tecnología Agraria y Alimentaria, Madrid, Spain; 2 Departamento de Biotecnología-Biología Vegetal, Escuela Técnica Superior de Ingeniería Agronómica, Alimentaria y de Biosistemas, Universidad Politécnica de Madrid, Madrid, Spain

**Keywords:** Plant immunity, Plant signalling, Plant stress responses

## Abstract

The success in the response of a plant to a pest depends on the regulatory networks that connect plant perception and plant response. Meta-analyses of transcriptomic responses are valuable tools to discover novel mechanisms in the plant/herbivore interplay. Considering the quantity and quality of available transcriptomic analyses, *Arabidopsis thaliana* was selected to test the ability of comprehensive meta-analyses to disentangle plant responses. The analysis of the transcriptomic data showed a general induction of biological processes commonly associated with the response to herbivory, like jasmonate signaling or glucosinolate biosynthesis. However, an uneven induction of many genes belonging to these biological categories was found, which was likely associated with the particularities of each specific Arabidopsis-herbivore interaction. A thorough analysis of the responses to the lepidopteran *Pieris rapae* and the spider mite *Tetranychus urticae* highlighted specificities in the perception and signaling pathways associated with the expression of receptors and transcription factors. This information was translated to a variable alteration of secondary metabolic pathways. In conclusion, transcriptomic meta-analysis has been revealed as a potent way to sort out relevant physiological processes in the plant response to herbivores. Translation of these transcriptomic-based analyses to crop species will permit a more appropriate design of biotechnological programs.

## Introduction

Plant evolution has led to the development of refined strategies of defense to perceive the attack of arthropod herbivores and display an appropriate defensive response^1,2^. However, induced responses are potentially complicated to understand because plants may be infested by generalist or specialist species, with various feeding modes (chewing, piercing, or sucking herbivores), from different developmental stages (from eggs to adults), and with distinct potential endosymbionts. Consequently, arthropod herbivores can elicit different types of defense responses^3–5^.

Plant defense response to herbivores is triggered by the activation of receptors that recognize conserved molecular patterns or specific molecules of attackers. In this recognition, there are two branches. One involves the use of transmembrane pattern recognition receptors (PRRs) that respond to herbivore- or damaged-associated molecular patterns (HAMPs or DAMPs), triggering an ordered sequence of molecular events which is called PAMP-triggered immunity (PTI)^6–8^. The other branch includes the use of intracellular receptors that identify pathogen virulence molecules, known as effectors, activating the effector-triggered immunity (ETI), an amplified response of the PTI^9^. In this signaling process, the molecular events displayed include the rapid activation of specific ion channels, the production of reactive oxygen species (ROS), and the induction of specific kinase cascades.

As a consequence, gene expression is altered by differential regulation of transcription factors (TFs). This transcriptomic reprogramming is predominantly coordinated by the interactions and crosstalk between the jasmonic acid (JA), ethylene (ET), and salicylic acid (SA) hormones^10,11^. Typically, the JA pathway is activated in response to chewing-biting herbivores and cell-content feeders, and the SA pathway is turned on against piercing-sucking insects^12^. The most relevant TF families in innate immune responses are the AP2/ERF, bHLH, MYB, NAC, WRKY, and bZIP families^13^. Numerous findings support the direct relationship between hormonal signals and TF functionality in *Arabidopsis thaliana*. SA signaling triggers the translocation of the regulatory protein NPR1 to the nucleus. In the nucleus, NPR1 interacts with members of the TGA subclass of the bZIP family that bind to the promoters of SA-responsive genes^14^. JA induces the degradation of the JAZ family of regulatory proteins. The elimination of JAZ proteins permits the activation of two branches in the induction of JA-responsive genes^15,16^. The MYC-branch is primarily controlled by the bHLH TFs MYC2, MYC3 and MYC4^17^. The ERF-branch is synergistically regulated by the ET-signaling pathway and leads to the activation of responsive genes controlled by the AP2/ERF TFs ORA59 and ERF1^18,19^.

Alterations in the expression of TFs change the accumulation patterns of several defensive proteins and of numerous enzymes involved in the biosynthesis of secondary metabolites. Defense-related proteins, such as chitinases, cysteine proteases or lectins, may have toxic or anti-nutritional effects^4^. Secondary metabolites contribute to plant immunity as bioactive toxic compounds or as volatiles attracting herbivore natural enemies^4^. For example, TFs of the MYC and MYB families regulate the expression of several antiherbivore genes, including terpenoids and flavonoids. MYC2 regulates sesquiterpene biosynthesis by binding the promoters of *TPS11* and *TPS21*^20^. *MYB75* overexpression lines showed reduced levels of kaempferol 3,7-dirhamnoside, which correlates with increased *P. brassicae* performance^21^. Besides, mutations in genes involved in the glucosinolate metabolism or regulation render plants highly susceptible to herbivory. TGG1 and TGG2 are two myrosinase enzymes that degrade glucosinolates to produce toxins. Weight gain of two Lepidoptera, *Trichoplusia ni* and *Manduca sexta*, was significantly increased on *tgg1tgg2* double mutants^22^.

Taken together, defensive signal transduction leads to a concerted regulation of downstream stress-responsive genes. This work is focused on the meta-analysis of the plant transcriptomic responses to herbivore stresses to determine specificities and commonalities. Starting from a set of 28 Arabidopsis-herbivore transcriptomic experiments, we performed a comprehensive analysis of the biological processes involving induced or repressed genes. From these results, a broad range of particularities in the plant response to diverse herbivores was revealed.

## Materials and methods

### Gene ontology enrichment analyses

In a previous work, a set of 28 Arabidopsis-herbivore transcriptomic experiments was collected and analyzed to obtain lists of differential expressed genes (DEGs)^23^. The selected experiments included a broad spectrum of diverse arthropods feeding on Arabidopsis plants of the ecotype Columbia-0 with a preferably vegetative stage of 4 weeks when infested. This starting information is compiled in the Supplementary Dataset S1. Processing and normalization of transcriptomic data were previously described in the Methods S1 file of Santamaria et al.^23^. Because of the different nature of the data, results of RNA-seq and microarray experiments were compared after extraction of gene expression and filtering by log2FC > 1.5 and p-value adjusted < 0.05, establishing similar thresholds for the different analyses. The list of DEGs for each experiment is included in the Supplementary Dataset S1. These data were used to search the Gene Ontology database employing the Metascape tool (http://metascape.org)^24^. Metascape uses the hypergeometric test and the Benjamini–Hochberg p-value correction algorithm to find the ontology terms present in a statistically greater number of genes than expected by chance. Each gene is examined for its pathway and process enrichment score making use of the Gene Ontology^25^, KEGG^26^, and other platforms. According to their pathways, genes are clustered in non-redundant clusters. A 0.3 kappa score was applied as the threshold to cast the tree into term clusters. Heatmap was depicted using the ClustVis tool^27^.

To explore the contribution of each experiment to the enrichment of GO ontologies, searches using keywords related to jasmonic acid, immune response, salicylic acid, chitin, and indole glucosinolate categories were performed in the Gene Ontology database using the AmiGO 2 project^28^. Obtained GO identifiers were used to collect the list of Arabidopsis genes by the BioMart tool in the Ensembl Plants section of the Ensembl Genomes platform^29,30^. Gene enrichment analyses were performed with the Bonferroni step-down test using ClueGO package^31^ in Cytoscape^32^.

### Expression analyses of gene families involved in different steps of the plant response

Several lists of genes were obtained from public repositories. For the generation of the receptor gene list, two platforms were searched, the Plant Resistance Genes Database 3.0 (PRGdb; http://prgdb.org), an open and updated database of pathogen recognition genes^33^, and the resistance gene analogs (RGAs) lists of genes^34^. Once the lists were compared and extracted, receptors were classified by the domains they present, differentiating between transmembrane and non-transmembrane. Lists of calcium sensor genes were obtained from literature^35–37^. The full set of Arabidopsis transcription factors (TFs) was downloaded from the Plant Transcription Factor Database (PlantTFDB v5.0) included in the plant regulatory data and analysis platform PlantRegMap^38^. Arabidopsis genes belonging to pathogenesis-related families were extracted from literature^39^. Comparison of total DEGs across selected experiments was conducted using Instant Clue software^40^, which performs a hierarchical clustering to classify the experiments and generates a heatmap for the visualization of the similar patterns of DEGs. Gene molecular networks were based in the protein–protein interactions generated in the STRING database version 11.0^41^. Venn diagrams were performed using the Venny 2.1 tool (Oliveros, J.C., 2007–2015, https://bioinfogp.cnb.csic.es/tools/venny/index.html).

### Metabolic databases

The KEGG (Kyoto Encyclopedia of Genes and Genomes) database was primarily managed to identify genes related to metabolic pathways^26^. Identified genes were classified in metabolic functional categories by using the Pathway Tools Omics Dashboard^42^. Schematic representations of metabolic pathways were based on the experimentally supported and computationally predicted metabolic pathways available in the AraCyc v16.0 database (http://pmn.plantcyc.org/ARA/class-instances?object=Pathways).

## Results

### Gene ontology term enrichment analysis

**Figure 1:**
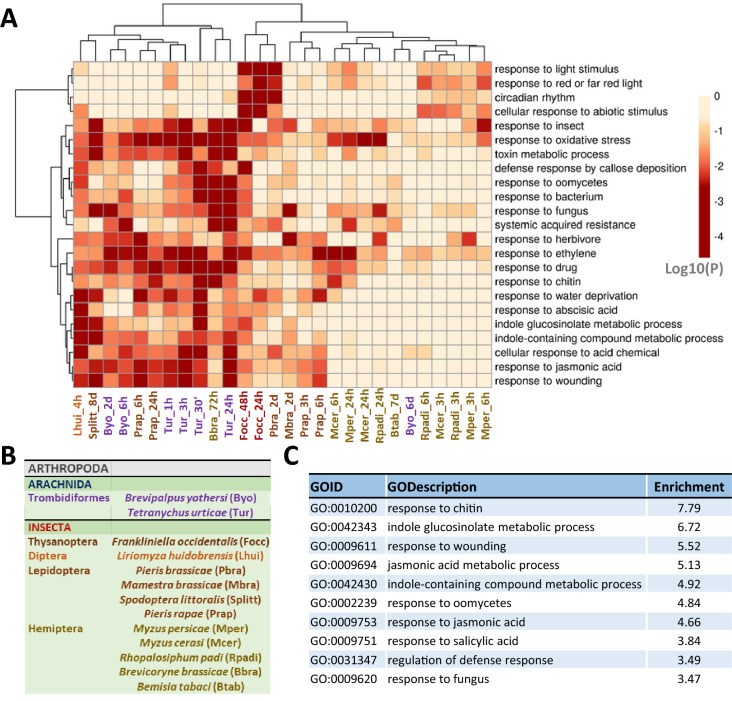
Meta-analysis of the transcriptomics experiments. (**A**) Heatmap showing the significance of the most enriched biological ontologies. (**B**) Taxonomic classification of the arthropods used in the experiments. (**C**) List of the top 10 enriched biological processes obtained from a single list of all the DEGs merged together.

A former analysis of 28 Arabidopsis-herbivore transcriptomic experiments reported a group of DEGs in the plant with a correlated expression^23^. To complement the information provided by correlated genes, a gene ontology enrichment analysis of the DEGs of this set of experiments was performed by the Metascape tool. The goal of this analysis was to detect the most affected biological processes. The top enriched biological processes and their enrichment patterns across the experiments were represented as a clustered heatmap (Fig. 1A). The results were not far from expected. The heatmap showed that DEGs were enriched in biological processes related to the plant response to other organisms, hormones and chitin, or involved in the biosynthesis of indole glucosinolates. However, not all the biological processes were enriched in all the experiments, with a bias related to taxonomy (Fig. 1B). For example, several biological process GO terms were not enriched upon aphid infestation. Likewise, a varied pattern was observed for the biological processes of circadian rhythm and response to light stimulus. The heatmap was complemented by a list of the top 10 enriched terms (Fig. 1C). These terms were obtained from a single list of all the obtained DEGs. The results were quite similar to those from the previous analysis, with the inclusion of the term “response to salicylic acid”. All the terms are related to the response of plants to biotic stresses.

### Analysis of defense-related categories

Based on the enriched gene ontologies found in the previous analyses, several terms related to plant defense were used to explore common/specific responses. Browsing the Gene Ontology (GO) database with jasmonic acid (JA), salicylic acid (SA)/immune response, chitin, and indole glucosinolate (IG) terms, GO identifiers related to these categories were retrieved. Besides, a list of Arabidopsis genes associated with these GO identifiers was constructed. When the global list of DEGs was searched, 166 genes related to jasmonic acid response, 173 genes related to salicylic acid/immune response, 113 genes related to chitin response, and 100 genes related to indole glucosinolate metabolism were found (Supplementary Dataset S1). Heatmaps were obtained to visualize the expression patterns (Supplementary Fig. S1). The highest number of induced genes was generally observed in the *T. urticae*, *Lyriomyza huidobrensis* and *Pieris rapae* experiments, followed by the experiments using *Brevipalpus yothersi* and *Spodoptera littoralis*.

Defense responses are closely associated with the recognition of the stress and altered gene expression. Therefore, DEGs related to these processes were identified (Supplementary Dataset S2). To analyze the molecular perception, a list of putative Arabidopsis gene receptors was created by extracting genes from databases and literature. The gene list included both transmembrane and intracellular receptors, for the triggering of the PTI and ETI, respectively. The final list was composed of 739 Arabidopsis gene receptors classified in different gene families based on the domains they contain. Cell-surface pattern-recognition receptors (PRRs) perceive diverse signals and stimuli from the environment and include 53 receptor-like proteins (RLP), 403 receptor-like kinases (RLKs), and 58 receptor-like cytoplasmic kinases (RLCKs). Besides, most disease resistance genes (R-genes) encode intracellular proteins with nucleotide binding-leucine-rich repeat (NBS-LRR) domains (also known as NLRs). These proteins differ primarily at the N-terminal domain and include 88 proteins with a Toll-like receptor (TIR), 39 proteins with a coiled-coil domain (CC), and 98 proteins with a diverse structure, presenting alternative combinations of CC, TIR, NBS, LRR, and other domains. Finally, calcium sensor proteins are represented by three main families, the calcineurin-B-like proteins and calcineurin-B-like interacting protein kinases (CBLs and CIPKs), the calmodulin (CaM) and calmodulin-like proteins (CMLs), and the calcium-dependent and calcium-related protein kinases (CPKs and CRKs). Transcription factors were identified and categorized using the full set of Arabidopsis TFs available in the Plant Transcription Factor Database (PlantTFDB). Depicted heatmaps were constructed using the 172 PRR genes, 361 NLR receptors, 62 calcium sensors, and 687 TFs differentially expressed in at least one experiment (Supplementary Fig. S1). As expected, the highest number of DEGs from these categories were found in the *T. urticae*, *L. huidobrensis*, and *P. rapae* experiments, which agree with their more pronounced response to the stress.

### Comparative analysis of the *T. urticae* and *P. rapae* experiments

From the array of previous results, the *T. urticae* and *P. rapae* experiments were selected to an in-depth comparison. This selection was based on the similar technology used (RNA-seq), the pronounced response of the plant to both species, and the possibility to compare results from four different time points at the first 24 h upon infestation (Supplementary Dataset S3).

**Figure 2:**
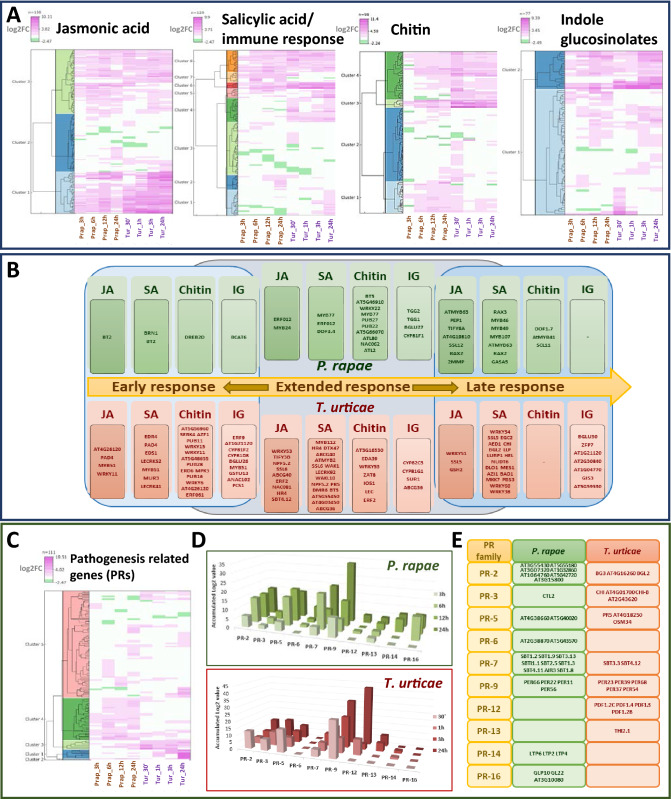
Comparison of the transcriptomics data at four time points of infestation by *P. rapae* or *T. urticae*. (**A**) Heatmaps showing the transcriptomic profile of the DEGs belonging to defence-related categories and detected at least in one experiment. (**B**) Schematic representation of the species-specific DEGs along plant response. (**C**) Heatmap showing the transcriptomic profile of the differentially expressed pathogenesis related genes detected at least in one experiment. (**D**) Accumulated expression of the DEGs from the PR categories at the different time points analysed. (**E**) Summary of the individual species-specific differentially expressed PR genes upon 24 h infestation.

Heatmaps were performed showing the DEGs found in at least one *T. urticae* or *P. rapae* experiment for the defense-related categories previously tested (Fig. 2A). The general expression pattern of JA associated genes was quite similar between *T. urticae* and *P. rapae* experiments. However, clustering highlighted groups of genes with a different expression pattern in the salicylic acid/immune response, chitin, and indole glucosinolate categories. A remarkable higher induction throughout *P. rapae* infestation was shown by genes of cluster 7 (SA/immune response) and cluster 1 (chitin) as well as a specific induction of genes in cluster 2 (SA/immune response) upon 24 h treatment. Likewise, groups of genes from cluster 4 (SA/immune response and chitin) were differentially expressed throughout *T. urticae* infestation, and genes of cluster 1 (SA/immune response) were specifically up-regulated upon 24 h mite treatment. Otherwise, specific mite induction of several genes of cluster 3 (SA/immune response) and cluster 2 (chitin) was only evident upon 30 min infestation. Scattered differential expression was found for the IG category, with several genes differentially up-regulated in both herbivore species.

Focusing on individual genes, the number of specifically up-regulated genes was much more elevated in *T. urticae* than in *P. rapae* at the earliest infestation time points reported for each species, 30 min for *T. urticae* and 3 h for *P. rapae*. Similarly, it was found in *T. urticae* a higher number of genes for the JA and SA categories with an extended up-regulation from the earliest time points to the latest time point reported, 24 h for both species (Fig. 2B). At the latest infestation time points, more genes included in the chitin category were up-regulated in *P. rapae* and a higher number of genes for the IG category were induced in *T. urticae*. Interestingly, many of the genes with a species-specific expression pattern were signal receptors and TFs. Receptor-like kinases from different families, such as the lectin-like (*LecRKS2*, *LecRK41*, *LecRK92*) and the wall-associated (*WAK1*, *WAKL10*), were up-regulated by a mite infestation. TFs belonging to the WRKY, MYB, NAC, and ERF families were specifically induced upon both *P. rapae* or *T. urticae* infestation. At the late response, whereas TFs of the MYB family were only found for *P. rapae* (*AtMYB41*, *AtMYB63*, *MYB46*, *MYB49*, *MYB107*), WRKY TFs were exclusive for *T. urticae* (*WRKY38*, *WRKY51*, *WRKY54*, *WRKY60*).

Differences between the expression patterns in both species were also evident when the up- or down-regulation of pathogenesis-related (PR) genes was shown in a heatmap (Fig. 2C). Most genes exhibited a species-specific pattern, with a substantial number of up-regulated genes of the PR-2, PR-7, and PR-16 families in the cluster 4 for *P. rapae* and of the PR-12 family in the cluster 1 for *T. urticae*. Besides, a few genes from cluster 2 appear up-regulated in both species. Focusing on the particular families, an analysis of the accumulated expression of DEGs along time points revealed the expression of many PR families from the earliest time point upon infestation and an elevated accumulation for some families at later time points. Differences between species were relevant for the expression of genes from some PR families, with an unequal up-regulation of genes from the PR-7 (subtilisins), PR-14 (lipid transfer proteins), and PR-16 (germins) families upon *P. rapae* infestation and the PR-3 (chitinases) and PR-12 (defensins) families upon *T. urticae* stress (Fig. 2D, E).

### Receptors and transcription factors in the *T. urticae* and *P. rapae* experiments

The large differences in the induced expression of genes in defense-related categories lead us to deep into the alterations in the expression patterns of plant receptors and TFs caused by *P. rapae* or *T. urticae* infestation.

**Figure 3:**
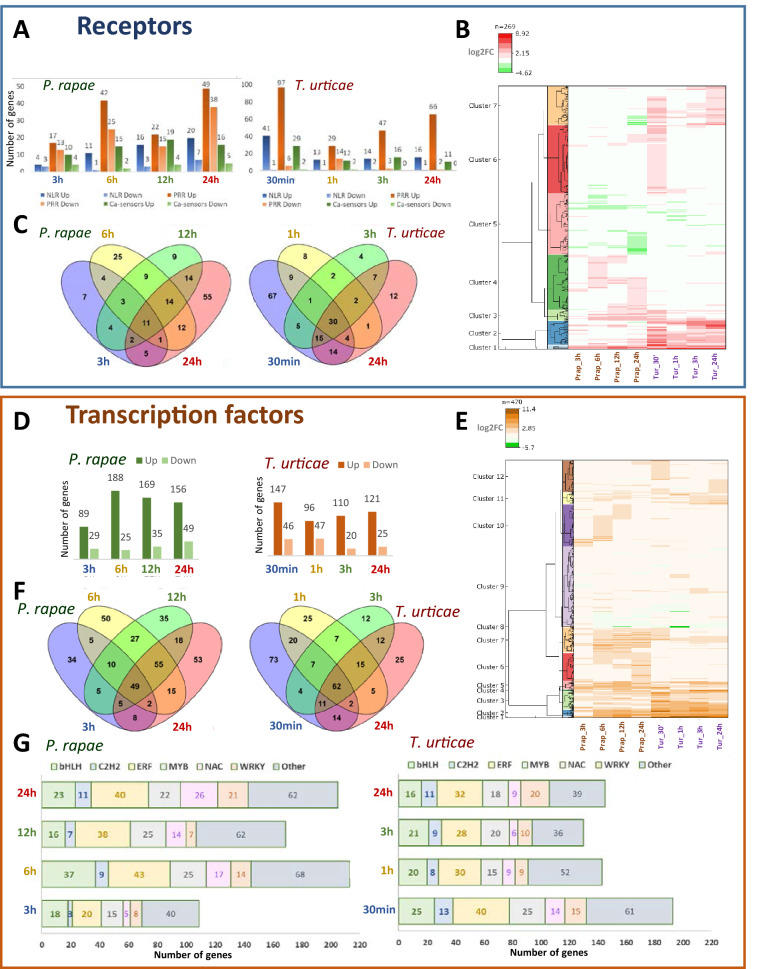
Comparison of the transcriptomics data for putative receptors and TFs at four time points of infestation by *P. rapae* or *T. urticae*. (**A**) Bars diagrams showing the number of up- or down-regulated receptors at each infestation time point. (**B**) Heatmap showing the transcriptomic profile of the differentially expressed receptors detected at least in one experiment. (**C**) Venn diagrams showing the number of specific and shared regulated receptors among infestation time points. (**D**) Bars diagrams showing the number of up- or down-regulated TFs at each infestation time point. (**E**) Heatmaps showing the transcriptomic profile of the differentially expressed TFs detected at least in one experiment. (**F**) Venn diagrams showing the number of specific and shared regulated TFs among infestation time points. (**G**) Bars diagrams showing the number of regulated TFs from the most relevant categories at each infestation time point.

When PRR and NLR receptors and Ca-sensors were analyzed, a characteristic pattern arose (Fig. 3A). In response to *P. rapae*, the number of DEGs trends to increase along time, from 51 DEGs at 3 h to 135 DEGs at 24 h, with a proportion of down-regulated genes around 30–40% at the four time points. In contrast, the highest number of DEGs was found at 30 min of *T. urticae* infestation. This number dropped from 176 DEGs at 30 min to 71 DEGs at 1 h and increased again up to 96 DEGs at 24 h. Likewise, the proportion of up- and down-regulated genes followed a different pattern. Whereas a maximum of 24% down-regulated genes was found at 1 h, the proportion of down-regulated genes was lower than 6% at 30 min, 3 h, and 24 h. The heatmap comparing the expression of these genes aids to explain this distinctive pattern (Fig. 3B). Whereas a group of genes in cluster 6 was specifically up-regulated upon 30 min *T. urticae* infestation, a stronger down-regulation was found in cluster 5 at 24 h *P. rapae* infestation. Besides, groups of up-regulated genes were found in cluster 4 specifically at 6 h or 24 h of *P. rapae* treatment. These expression patterns were highlighted in the Venn diagrams (Fig. 3C). The highest numbers of DEGs specific of a time point appeared at 6 h and 24 h of *P. rapae* infestation. Conversely, a strongly elevated number of DEGs was found at 30 min of *T. urticae* infestation.

The expression patterns of TFs were also different between *T. urticae* and *P. rapae* experiments. At the earliest time point, *P. rapae* had the lowest number of differentially expressed TFs, while the highest number was recovered for *T. urticae* (Fig. 3D). Moreover, whereas for *P. rapae* the proportion of down-regulated TFs decreased in the next time point, it increased for *T. urticae*. Once more, differences were highlighted in a heatmap showing the expression patterns of all the differentially expressed TFs (Fig. 3E). Groups of TFs specifically up-regulated in *P. rapae* experiments appeared in cluster 10 at 3 h, 6 h or 12 h, and in cluster 6 at 24 h, with a remarkable induction of members of the MYB and NAC families. For *T. urticae*, small groups of TFs for the 1 h and 3 h experiments were found in cluster 9. Larger groups of TFs specifically expressed at 30 min or 24 h were found in cluster 12, including a notable representation of members from the ERF and WRKY families. Furthermore, a set of TFs differentially expressed in most experiments englobes clusters 1 to 5. Again, Venn diagrams highlighted these expression patterns (Fig. 3F). For *P. rapae* experiments, a considerable number of specific DEGs were detected for each time point, together with an elevated number of TFs differentially expressed in the four time points or in the latest three time points. For *T. urticae*, the highest number of differentially expressed TFs specific for a time point was found at 30 min, and a considerable number of TFs were differentially expressed in the four time points. When focused on specific TF families, many DEGs were classified in the WRKY, NAC, MYB, ERF, C2H2, and bHLH families (Fig. 3G). When the number of members was compared along with time points, the lowest number of DEGs for any family was found at 3 h for *P. rapae*. After this time point, the members of the ERF family decreased from 6 to 24 h, and those of the NAC and WRKY families increased. Regarding *T. urticae* experiments, the highest number of DEGs for any family was found at 30 min except for the WRKY family, which had its maximum at 24 h.

**Figure 4:**
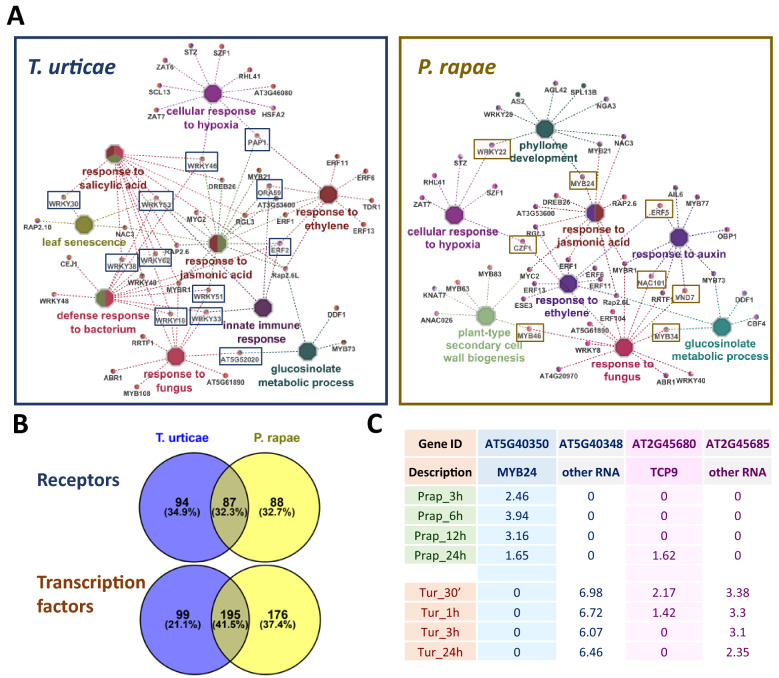
Comparison of putative receptors and TFs differentially regulated in response to *P. rapae* or *T. urticae*. (**A**) Networks showing the String-based interactions after five rounds of adding nodes to the initial TFs detected in the defense-related categories and distinctive of the Arabidopsis response to *T. urticae* and *P. rapae* (boxed). (**B**) Venn diagrams showing the number of specific and shared regulated receptors and TFs. (**C**) Table showing the expression of two TFs regulated by lncRNAs.

To ascertain the pathways controlled by the induced TFs, enrichments of biological processes were determined for the TFs differentially expressed DEG with a fold change higher than 2.5 at least in one time-point (Fig. 4A). Enrichments of responses related to defense mechanisms were predominantly found. Responses to the defense-related hormones JA and ethylene were shared, as well as the response to fungus, the cellular response to hypoxia, and the glucosinolate metabolic process. Responses to salicylic acid and bacteria were uniquely determined for *T. urticae*. Regarding responses related to plant growth and development, most enriched processes were induced by *P. rapae*, including the response to auxins, the phyllome development, and the secondary cell-wall biogenesis. Differences in enriched processes are concomitant to a large number of induced TFs with a species-specific pattern (Fig. 4B). Interestingly, many TFs connecting enriched biological processes were species-specific (Fig. 4A). The broad variability in TF regulation was associated with a high number of receptors differentially regulated in response to *P. rapae* and *T. urticae* (Fig. 4B). The expression of around 50% of the receptors altered in a species was not modified in the other species. Some potential natural antisense genes for these TFs exhibited a diverging expression pattern after *P. rapae* or *T. urticae* infestation (Fig. 4C). Whereas the antisense for the JA-induced *MYB24* gene was highly up-regulated in *T. urticae* and was not regulated in *P. rapae*, the corresponding *MYB24* gene was up-regulated in *P. rapae* and not regulated in *T. urticae*. The JA-repressing TF *TCP9* was up-regulated uniquely at early time points upon *T. urticae* treatment as well as its potential antisense gene, which remains induced along with the whole treatment. In contrast, although the antisense gene of *TCP9* was not up-regulated, the *TCP9* TF was only induced upon 24 h of *P. rapae* infestation.

### Differential expressed genes involved in the secondary metabolism

**Figure 5:**
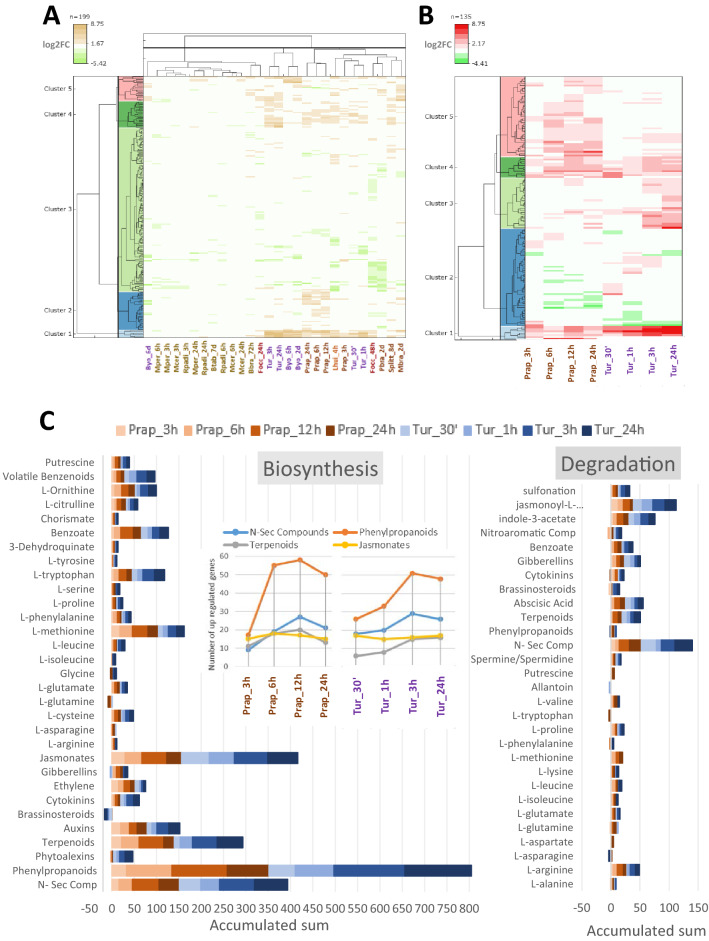
Analysis of the transcriptomic changes in the genes associated to secondary metabolic pathways. (**A**) Heatmap showing the transcriptomic profile of the DEGs belonging to secondary metabolism categories and detected at least in one experiment. (**B**) Heatmap restricted to the DEGs in *T. urticae* and *P. rapae* experiments. (**C**) Number of DEGs and their accumulated expression from the secondary metabolism categories at four time points of infestation by *P. rapae* or *T. urticae*.

Dissimilarities on the set of receptors and TFs with altered expression most likely lead to variations in the expression of genes involved in the production of secondary metabolites. The list of DEGs in at least one of the 28 selected experiments was searched to find genes stored in the KEGG database of metabolic pathways (Supplementary Dataset S4). The hierarchical clustering of the identified 199 DEGs distinguishes five clusters (Fig. 5A). Genes up-regulated in *T. urticae* experiments were mainly found in clusters 1, 4 and 5. In clusters 1 and 4 many DEGs were shared by plant responses to *T. urticae*, *P. rapae* and *L. huidobrensis*, to *B. yothersi* in cluster 1 and *S. littoralis* in cluster 4. In cluster 5, groups of induced genes were common to *T. urticae*, *B. yothersi* and *Mamestra brassicae* treatments. Cluster 2 was formed by DEGs mostly up-regulated in *P. rapae* experiments, and the large cluster 3 mainly included down-regulated genes identified in one or a few experiments. As the experiments with more than 50 up-regulated genes were those from *T. urticae* at 3 h and 24 h, and from *P. rapae* at 6 h and 24 h, the following analyses were focused again on the experiments involving these two species. Differences were highlighted in a heatmap showing the expression patterns of the 135 DEGs related to the secondary metabolism (Fig. 5B). Genes up-regulated in experiments with both species (clusters 1 and 4), and specifically induced for *P. rapae* and *T. urticae* were found (clusters 3 and 5). Cluster 2 was mainly formed by down-regulated genes, with some small groups of genes up-regulated in one species and down-regulated in the other species.

**Figure 6:**
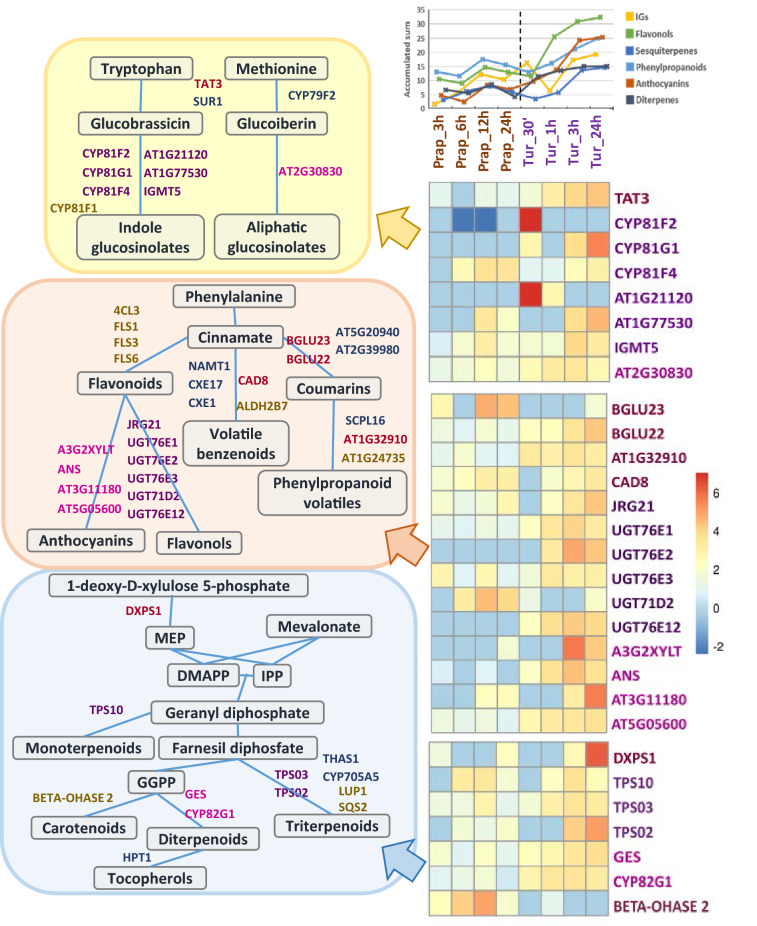
Analysis of secondary metabolic pathways upon *T. urticae* and *P. rapae* infestation. The left column shows schematic representations of the pathways leading to the synthesis of main secondary metabolites involved in plant defense. Colour names from pink-based palette identify enzymes involved in different steps or branches of a pathway. Green names correspond to enzymes exclusively up regulated upon *T. urticae* infestation. The right column englobes a linear graphic showing the accumulated expression of the DEGs involved in the synthesis of the main secondary compounds and heatmaps showing the relative expression in the pathway of every DEG with a fold change higher than four at least in one time-point.

To unveil particular altered pathways, RNA-Seq data were classified into metabolic functional categories by using the Pathway Tools Omics Dashboard (Supplementary Dataset S5). Similar patterns were detected when the sum of fold changes for DEGs was compared, with an evident trend to enhance biosynthetic pathways (Fig. 5C). Remarkably, the four categories with the highest accumulated values of gene up-regulation were those related to the biosynthesis of phenylpropanoids, nitrogen-containing secondary compounds, terpenoids and jasmonates. The number of induced genes involved in the biosynthesis of phenylpropanoids and nitrogen-containing secondary compounds was higher at the earliest time point upon *T. urticae* infestation and increased in response to both species (insert in Fig. 5C). Terpenoids genes showed a slight increase with minor variations. In contrast, the number of up-regulated genes related to jasmonate biosynthesis kept constant along time and was similar in both experiments. When DEGs with a fold change higher than four were selected, specific patterns regarding metabolic routes arisen (Fig. 6). From the genes related to the production of glucosinolates, a stronger induction was detected in the *T. urticae* experiments, with a remarkable early up-regulation of genes involved in the synthesis of indole glucosinolates. Regarding the phenylpropanoid pathway, besides specificities in the expression of genes involved in the production of coumarins or flavonols, enhanced induction of the genes involved in the biosynthesis of anthocyanins was found for *T. urticae*. Finally, whereas a positive trend in the production of diterpenoids was apparent in *T. urticae* experiments, *P. rapae* showed a trend to induce the synthesis of carotenoids.

## Discussion

Combined species-specific and meta-analysis of transcriptomic responses permitted identify novel relevant processes and specificities in the Arabidopsis response to the spider mite *T. urticae*^23^. This dataset offers the possibility to increase knowledge on the response of the plant to a variety of arthropod herbivores. Several questions were set out: Does the plant response depend on the type of arthropod or feeding guild? Are the same genes from common signaling pathways induced or a certain variability may be found depending on the perception of the attacker? Are the common signaling pathways producing a similar set of defensive compounds?

### Does the plant response depend on the type of arthropod or feeding guild?

Traditionally, the JA pathway has been associated with responses to chewing-biting herbivores and cell-content feeders, and the SA pathway against piercing-sucking insects. However, the JA pathway also modulates the responses to phloem-feeding insects^43^. Likewise, the SA-regulated defenses are involved in the responses to non-sucking arthropods as the mite *T. urticae*^44^. Two approaches were performed to explore the relationship between plant responses and feeding guild.

The Metascape analysis revealed the shared deregulation of plant responses by chewers and cell-sucking feeders, including mites, thrips, lepidopteran, and dipteran species. Most enriched categories were closely associated with the jasmonate signaling pathway and have been broadly associated with biotic stresses. For example, the production of indole glucosinolates occurs in response to many biotic attacks, being secondary metabolites toxic to a range of microorganisms, nematodes, and insects^45^. Likewise, the chitin of phytopathogenic fungi, nematodes, and arthropods is recognized by the plant, activating innate or adaptive plant defense responses^46^. Enhanced protection against herbivores has been suggested as a general response to perception of chitin^47^. Furthermore, several biological processes were broadly distinctive of the hemipteran feeding guild. Circadian rhythms and light responses exhibited high deregulation by pierce-sucking insects, which is in line with earlier studies on the central role of the circadian clock gene *CCA-1*^48^. Whereas loss-of-function mutants were more susceptible to aphid infestation, arrhythmic *CCA-1* overexpressors exhibited enhanced resistance concomitant to a positive regulation on the biosynthesis of indole glucosinolates. Interestingly, these enriched processes were not strictly associated with the feeding guild. For example, the thrip *Frankliniella occidentalis* induced light and circadian rhythm responses. Moreover, the plant response to the aphid *Brevicoryne brassicae* was more similar to that detected for chewing and cell-content feeders, which agree with previous results showing that SA signaling does not have a significant role in mediating plant defenses against this aphid^49^.

The second approach was based on the ontology terms associated with each DEG. The transcriptomics-derived heatmaps globally corroborate Metascape analyses. Chewing and cell-content feeders triggered a stronger alteration in the expression of JA, SA, chitin, and IG related genes than pierce-sucking insects. The most remarkable differences were observed in the JA and SA associated responses. Whereas most JA- and many SA-related genes were specially regulated in chewing and cell-content feeders, several genes associated with a positive regulation of the SA response were preferentially altered in aphids and mites. Examples included a group of WRKY TFs negatively affecting JA responses. Silencing mutants of *WRKY51* and *WRKY62* failed to suppress the induction of JA-responsive genes^50,51^. Besides, in the *wrky54wrky70* double mutant, the SA and JA/ET responsive genes *PR1* and *PDF1.2* were up-regulated^52^.

### Are the same genes from common signaling pathways induced or a certain variability may be found depending on the perception of the attacker?

A comparison of the four-time RNA-seq experiments with *T. urticae* and *P. rapae* was considered the best way to determine differences at the gene level on common signaling pathways. Enhanced enrichment of closely related biological processes associated with herbivory, such as JA, SA, and chitin responses or IG metabolism, was previously reported for both *P. rapae*^53,54^ and *T. urticae*^44^. These processes are triggered by the molecular perception of the attacker and are dependent on the transcriptional rewiring of the gene expression profiles mediated by TFs. The ultimate consequence of all these molecular rearrangements will rely on the capacity to produce secondary metabolites and defense proteins with a deleterious effect on the attacker, or volatile compounds to attract natural enemies of the herbivore^4,55^. Therefore, differences in the regulation of certain specific genes associated with a biological process will be translated into a more efficient defense against the actual herbivore species. Particularities in common processes depend likely on specificities in the perception of the herbivore attacker. Fine-tuning of receptor networks by species-specific particularities will affect all the associated pathways, from the early transmission of the signals to the final rearrangement of the secondary metabolism^56^. The dynamical comparison of the Arabidopsis responses to *P. rapae* and *T. urticae* supports this hypothesis. The central role of JA signaling was modulated by a substantial variation in the expression of intracellular and extracellular receptors, translated into differential regulation of a great set of JA-related TFs. The relevance on the plasticity in the regulation of TFs should be linked to the role of JA in a variety of pathways leading to the synthesis of secondary metabolites with a known anti-herbivore functionality, such as anthocyanins, terpenoids, glucosinolates, alkaloids, or flavonoids^57,58^. Likewise, the relationship between JA and the increment of proteins from the PR-3, 4, and 12 families has been largely documented^59^. Consequently, although most PR families and secondary metabolism categories were affected by both herbivore species, meaningful specificities were found when focusing on the expression of particular genes.

### Are the common signaling pathways producing a similar set of defensive compounds?

Despite the potential variability in the final plant response to herbivore stress, most omics analyses lack a detailed dissection of the proteins and enzymes acting as direct defenses or involved in the production of toxic compounds. Many studies use the expression of the genes encoding the PR proteins VSP1 and 2, PDF1.2, or PR1 and 2, to associate the defensive response and the hormonal signaling pathways^54,60,61^. Likewise, the association of specific metabolic routes in the response to an herbivore attack has been reduced to particular genes. For example, the *TPS4* gene, which encodes a geranyllinalool synthase involved in the production of terpenes, is induced by *P. rapae*^54^. Again, a comparison of the dynamic response to *P. rapae* and *T. urticae* offered a global and more accurate vision of the specificities in the production of both PR proteins and potentially toxic secondary metabolites. Defense proteins from many PR families were up-regulated upon both treatments, but this regulation was uneven in terms of specific families and members. Germin-like proteins (PR-16), lipid transfer proteins (PR-14), and proteinase inhibitors (PR-6) were relevant in the response to *P. rapae*, and defensins (PR-12) were induced upon *T. urticae* attack. Besides, specificities in the up-regulation of members from commonly regulated PR families were found. For example, up-regulated members of the PR-7 family, subtilisins, have been associated with disparate biological processes. Whereas the subtilisin proteins SBT3.3 and SBT4.12 induced by *T. urticae* were previously related to the immune priming or the response to jasmonate^62,63^, the most differentially induced subtilisins to *P. rapae*, SBTI1.1 and SBT1.2, participate in the regulation of water use efficiency by modulating stomatal density or in the activation of phytosulfokines involved in growth and differentiation processes^64,65^. Similar findings were found in the transcriptional dynamics of genes involved in the production of secondary metabolites. A panoramic view of the biosynthesis and degradation categories displayed a widespread pattern of response against both herbivores. However, a more thorough inspection of individual routes and genes unveiled substantial distinctions. Spider mites induce several plant defense pathways but only some of them are effective. Among them, JA-regulated accumulation of secondary IG metabolites was found to affect the ability of the spider mite to use Arabidopsis as a host plant^44^. On the contrary, *P. rapae* is adapted to the glucosinolates produced by crucifers as chemical defenses^66^. In agreement with these findings, genes encoding enzymes for glucosinolate production were quick and highly induced upon *T. urticae* infestation. One of them, *SUR1*, has been determined as a key gene to favor indole glucosinolate production against auxin biosynthesis^67^. In consequence, Arabidopsis plants should activate additional effective defense responses to reduce the performance of *P. rapae* caterpillars. Genes involved in the production of flavonoids, phenylpropanoids, and phytoalexins, were upregulated upon *P. rapae* infestation^54^. These metabolites can function as deterrents or repellents or cause indirect defensive effects by their emission as volatile organic compounds to attract natural enemies of herbivores^68,69^. As plant volatile emission can vary with the herbivore and the plant host, the recruitment of the suited herbivore enemies depends on the up-regulation of the correct genes^70,71^. Up-regulation of genes involved in the production of volatile benzenoids, such as *PAL1* and *BMST1*, was formerly found in the response to both species^44,72^. Although some genes putatively involved in the synthesis of volatile benzenoids and phenylpropanoids were specifically up-regulated, large differences in the activation of these routes were not apparent. In contrast, there were pronounced differences in the expression patterns of various enzymes involved in the synthesis of anthocyanins and flavonols, predicting a key role of these compounds in the Arabidopsis defense against spider mites. Likewise, the up-regulation of genes required for the biosynthesis of terpenoids was commonly found in the plant response to *P. rapae* and *T. urticae*, including *TPS2*, *TPS3*, *TPS4*, *TPS10*, and *CYP82G1*^44,72^. Although the general pathway for the synthesis of terpenes was positively affected by both herbivores, several enzymes involved in the production of diterpenoids were strongly expressed upon *T. urticae* attack. TPS04/GES and CYP82G1 are involved in the synthesis of the herbivore-induced volatile TMTT, which influenced the predatory/spider mite relationship in lima bean leaves^73^. HPT1 is involved in the biosynthesis of tocopherols from homogentisic acid, which have a positive effect on the Arabidopsis defense against *Pseudomonas syringae* and *Botrytis cinerea*^74,75^. Variations in the biosynthetic pathways of triterpenoids were also found. *T. urticae* attack induced the expression of the enzymes THAS1 and CYP705A5. These proteins are involved in the production of thalianol-derived triterpenes, which have been implicated in the modulation of the root microbiota^76^. *LUP1* and *SQS2* were up-regulated upon *P. rapae* infestation. LUP1 participates in the synthesis of lupeol, a triterpene that causes cytoplasmic membrane damage of the protozoan parasite *Leishmania donovani*^77^. Besides, the higher induction of the carotenoid pathway by *P. rapae* could be associated with its reported ability to visually discriminating between green and variegated green-whitish plants^78^.

In conclusion, the in-depth meta-analysis of transcriptomic responses was successful to partially disentangle the Arabidopsis response to arthropod herbivory. Besides feeding guild as a major contributor, we found a broad set of genes likely involved in the particularities of the plant–herbivore interaction. A thorough analysis of the responses to *P. rapae* and *T. urticae* highlighted specificities in the perception and signaling pathways associated with the expression of receptors and TFs. Consequently, the information coming from these interconnected signaling pathways was translated to a variable alteration of secondary metabolic pathways. This strategy has been revealed as a potent way to sort out relevant processes in the plant response to herbivores. The next step would imply decoding the contribution of altered modules to the final response. Pushing up the databases and tools needed to properly construct a solid framework would promote an appropriate mathematical analysis to decipher hallmarks and key contributors. This information should be translated to crop species for a more appropriate and specific design of biotechnological programs.

## Supplementary Information

Supplementary Information 1.

Supplementary Information 2.

Supplementary Information 3.

Supplementary Information 4.

Supplementary Information 5.

Supplementary Information 6.

Supplementary caption.

## Data Availability

All data generated or analyzed during this study are included in this published article (and its Supplementary Information files).
