# Supplementary material for: Disentangling transcriptional responses in plant defense against arthropod herbivores
Source: Sci Rep. 2021 Jun 21;11:12996. doi: 10.1038/s41598-021-92468-6 (PMC8217245; doi:10.1038/s41598-021-92468-6)
Supplement: Supplementary file 6 — Supplementary Information 6. [file 41598_2021_92468_MOESM6_ESM.pdf]

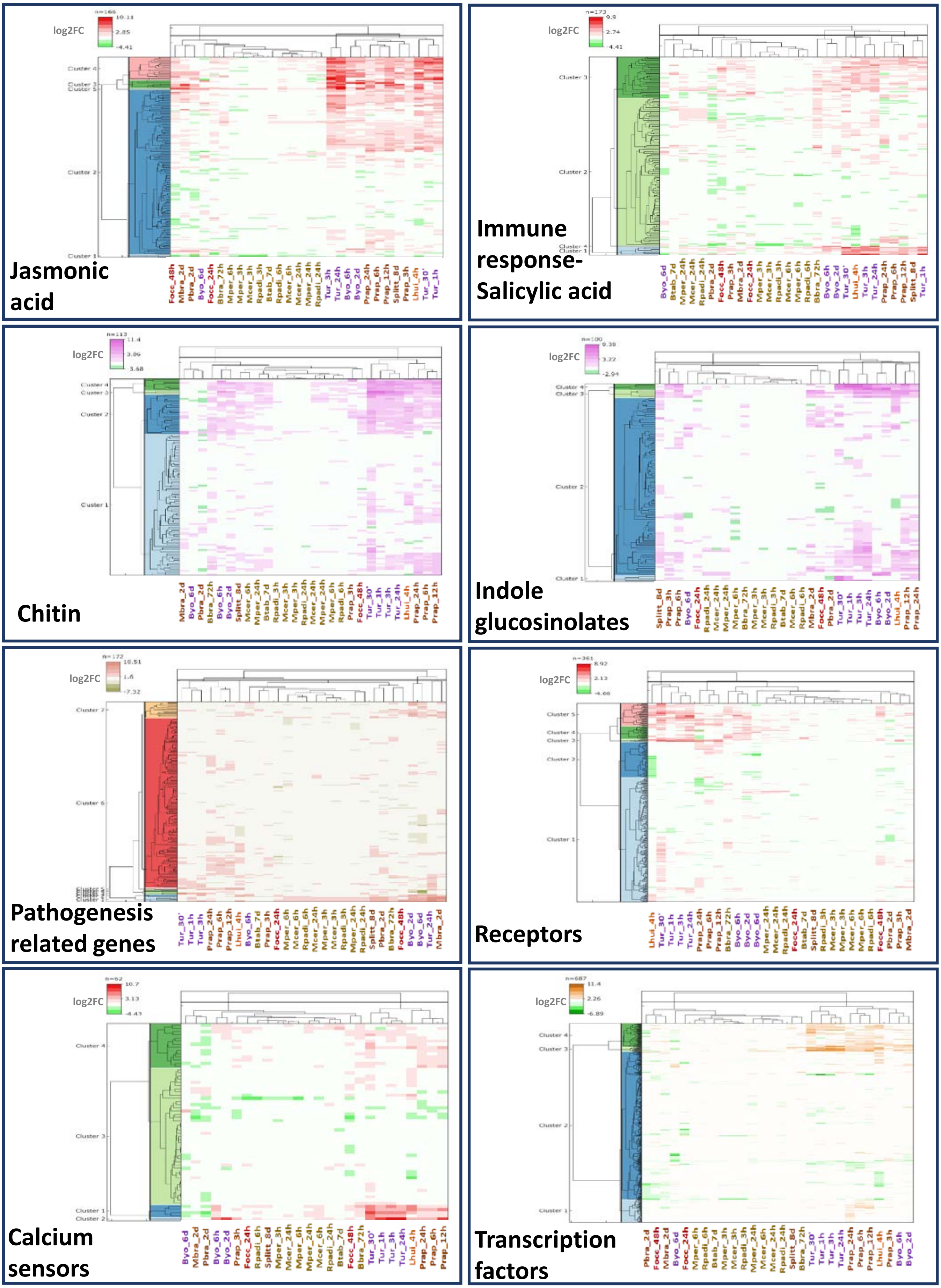

FIGURE S1. Heatmaps showing the transcriptomic profile of the DEGs belonging to different defence-related categories and detected at least in one experiment.
