# Supplementary material for: Disentangling transcriptional responses in plant defense against arthropod herbivores
Source: Sci Rep. 2021 Jun 21;11:12996. doi: 10.1038/s41598-021-92468-6 (PMC8217245; doi:10.1038/s41598-021-92468-6)
Supplement: Supplementary file 7 — Supplementary caption. [file 41598_2021_92468_MOESM7_ESM.docx]

**Supplementary information**

**Figure S1.** Heatmaps showing the transcriptomic profile of the DEGs belonging to different defense-related categories and detected at least in one experiment.

**Dataset S2.** Differentially expressed genes by functional categories in Arabidopsis upon infestation using different herbivores.

**Dataset S3.** Differentially expressed genes by functional categories in Arabidopsis upon infestation with *P. rapae* or *T. urticae*.

**Dataset S4.** Differentially expressed genes related to the metabolism of secondary compounds in Arabidopsis upon infestation using different herbivores.

**Dataset S5.** Differentially expressed genes related to the metabolism of secondary compounds in Arabidopsis upon infestation with *P. rapae* and *T. urticae*.
